# Case Report: CBFA2T3::GLIS2-positive myeloid sarcoma with focal bone marrow involvement mimicking Ewing sarcoma in an infant

**DOI:** 10.3389/fonc.2026.1936497

**Published:** 2026-09-17

**Authors:** Siman Chen, Jianxia Chen, Qiang Yao

**Affiliations:** 1 Clinical Laboratory, Longgang Central Hospital of Shenzhen, Shenzhen, China; 2 Department of Laboratory Medicine, Shenzhen Children’s Hospital, Affiliated to Shantou University Medical College, Shenzhen, Guangdong, China

**Keywords:** acute megakaryoblastic leukemia, CBFA2T3::GLIS2, Ewing sarcoma, megakaryoblastic differentiation, myeloid sarcoma, RNA sequencing, small round cell tumor

## Abstract

CBFA2T3::GLIS2-positive myeloid neoplasms occur predominantly in infants and can closely mimic pediatric small round cell sarcomas. We describe a 14-month-old boy who presented with left leg pain, an abnormal gait, and a destructive left ischial mass. Tissue biopsy was initially interpreted as an undifferentiated Ewing-like sarcoma. Bone marrow aspiration contained approximately 2% unclassified large cells, while trephine biopsy demonstrated focal tumor infiltration. Marrow tumor cells showed strong CD56 and weak CD61 expression and were positive for WT1, CD43, ERG, CD117, and BCL2. Lysozyme, MPO, TdT, and CD71 were negative. Diagnostic bone marrow flow cytometry was not performed. Molecular studies on pelvic lesion tissue showed negative results on EWSR1 break-apart fluorescence *in situ* hybridization (FISH). Broad RNA fusion testing detected CBFA2T3::GLIS2, joining CBFA2T3 exon 11 to GLIS2 exon 3, without a covered Ewing or Ewing-like sarcoma-defining fusion. After one cycle of cyclophosphamide, doxorubicin, and vincristine under the provisional sarcoma diagnosis, treatment was redirected to a 10-day course of cytarabine, etoposide, and daunorubicin. Severe myelosuppression was managed with anti-infective and supportive therapy. Subsequent marrow morphology, trephine biopsy, and flow cytometry showed no evident residual tumor. Follow-up MRI demonstrated marked reduction of the pelvic mass, although residual osseous abnormalities persisted. The patient subsequently continued treatment elsewhere. Available records documented additional targeted therapy and maternal haploidentical hematopoietic stem cell transplantation (HSCT). The first reported post-transplant molecular assessment detected no CBFA2T3::GLIS2 fusion transcript. Detailed post-transfer treatment parameters and long-term outcomes were unavailable. This case highlights the value of integrated tissue and marrow assessment and broad RNA fusion testing in EWSR1-negative infantile small round cell tumors.

## 1 Introduction

Myeloid sarcoma, historically termed chloroma or granulocytic sarcoma, is a tumor mass composed of myeloid blasts at an anatomical site other than the bone marrow (1–4). It may precede, accompany, or follow acute myeloid leukemia (AML). Diagnosis therefore requires integration of tissue morphology and immunophenotype with marrow findings and molecular genetics (1–4). When conventional myeloid markers are weak or absent, tissue-limited assessment may misclassify the lesion as an undifferentiated sarcoma or another small round cell tumor (3, 4).

CBFA2T3::GLIS2 arises from a cryptic chromosome 16 inversion and defines an aggressive pediatric AML subgroup enriched in non-Down syndrome acute megakaryoblastic leukemia (AMKL) (5, 6). The fusion occurs predominantly in infants and young children and is associated with induction resistance, frequent relapse, and poor survival (5–7). A recurrent immunophenotype termed the RAM phenotype (named after the initials of the index patient) features bright CD56, dim or absent CD45 and CD38, absenthuman leukocyte antigen-DR (HLA-DR), and variable CD34, CD33, and CD117 (8–11). This profile can resemble marrow metastasis from a non-hematopoietic tumor (8–11). Megakaryocytic glycoproteins, including CD41a, CD61, and CD42b, support AMKL lineage, although their expression may be heterogeneous or difficult to demonstrate in tissue (7, 12, 13).

Ewing sarcoma is an important differential diagnosis in children with destructive pelvic bone lesions and uniform small round cell morphology (14–17). CD99 and FLI-1 are sensitive but not lineage-specific, while NKX2.2 provides supportive rather than definitive evidence (14, 15). Molecular confirmation of an EWSR1::ETS or, less commonly, FUS::ETS fusion is therefore essential (16, 17). CIC-rearranged and BCOR-altered round cell sarcomas should also be considered when classic Ewing-associated testing is negative (16, 17). Broad RNA fusion sequencing is particularly valuable when morphology, immunohistochemistry, and targeted fluorescence *in situ* hybridization (FISH) are discordant (18). Recent reports describe CBFA2T3::GLIS2-positive myeloid sarcoma and extramedullary AMKL relapse mimicking Ewing sarcoma (12, 13). We report a diagnostically challenging *de novo* presentation in an infant with a pelvic mass and only focal marrow involvement.

## 2 Case description

### 2.1 Clinical data and imaging examinations

A 1-year-2-month-old boy was admitted on 2 November 2025 after more than 2 months of progressive left leg pain, gait abnormality, and a left pelvic mass. Synovitis had been initially considered at an outside hospital, but the symptoms did not improve. The child had no fever, weight loss, or other systemic symptoms. Physical examination showed a left-sided limp without swelling or tenderness of either hip; both lower limbs were symmetric in circumference.

Pelvic CT angiography (CTA) performed on 4 November 2025 demonstrated an ill-defined osteolytic lesion of the left ischium measuring approximately 22 mm × 32 mm × 41 mm (maximum axial dimensions × craniocaudal dimension). The marrow cavity was replaced by soft-tissue density, with cortical discontinuity, residual irregular osseous fragments, and a suspected lamellated periosteal reaction. A large adjacent soft-tissue mass extended both medially and laterally, predominantly medially, invaded the obturator internus region, and compressed or displaced the left obturator neurovascular structures. The lesion showed mild-to-moderate heterogeneous enhancement. The radiologic impression favored a malignant primary bone tumor, although osteomyelitis or bone abscess remained in the initial differential diagnosis.

Pelvic MRI performed on 5 November 2025 showed a heterogeneous left ischial mass measuring approximately 24 mm × 35 mm × 40 mm. The lesion was predominantly isointense to slightly hyperintense on T1-weighted images, hyperintense on T2-weighted and fat-suppressed sequences, and contained small internal low-signal foci. Marked diffusion restriction and prominent heterogeneous enhancement were present. The lesion involved the left ischial region and adjacent articular structures, with extensive edema and enhancement in surrounding muscles and soft tissues. Additional abnormal signals in the right ischium and bilateral iliac bones were considered suspicious for multifocal osseous involvement. The initial imaging differential diagnosis included Ewing sarcoma or another small round cell tumor, metastatic disease—particularly neuroblastoma in this age group—and Langerhans cell histiocytosis; osteomyelitis or bone abscess remained an important non-neoplastic mimic. Representative baseline CT and paired diagnostic/follow-up MRI images are shown in **Figures 1C, D**, respectively.

**Figure 1:**
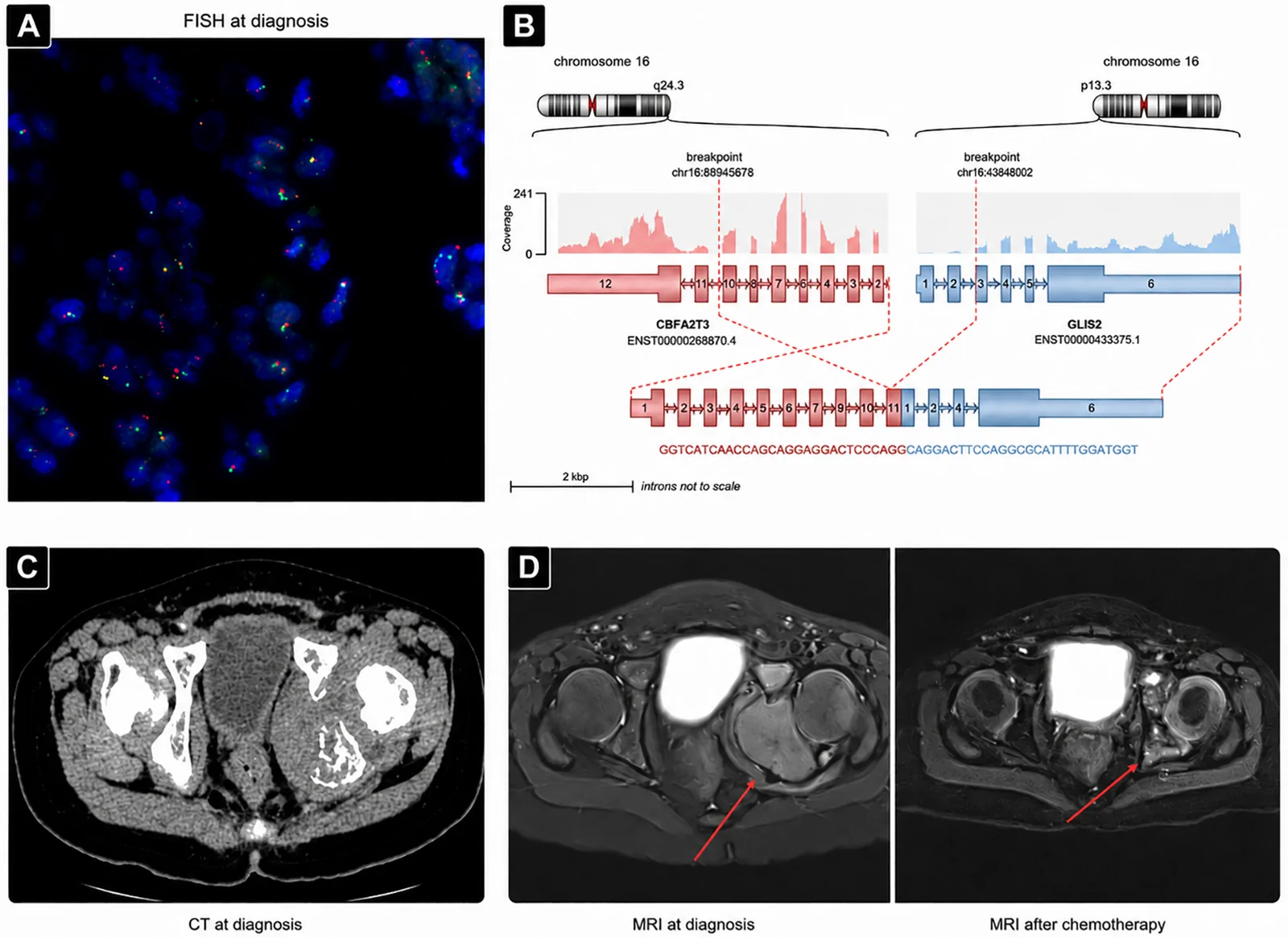
Molecular and radiologic findings. **(A)** EWSR1 break-apart fluorescence *in situ* hybridization performed on the pelvic lesion. Fused signals were observed in 91% of nuclei and split signals in 9%, below the laboratory cutoff of 15%; the result was interpreted as negative for EWSR1 rearrangement. **(B)** Schematic representation of the in-frame CBFA2T3::GLIS2 fusion identified by RNA sequencing, joining CBFA2T3 exon 11 to GLIS2 exon 3. **(C)** Baseline pelvic CT showing the destructive osteolytic left ischial lesion, cortical disruption, irregular residual osseous fragments/periosteal reaction, and a large adjacent soft-tissue component. **(D)** Paired pelvic MRI images at diagnosis and after myeloid-directed chemotherapy showing marked reduction of the soft-tissue mass and diffusion restriction, with persistent residual osseous destruction and enhancement.

The chronological sequence of the patient’s presentation, diagnostic investigations, treatment, and follow-up is summarized in **Table 1**.

**Table 1:** Timeline of clinical events, diagnostic investigations, and management.

Date/period	Key clinical event	Diagnostic findings	Management
Approximately September 2025	Progressive left leg pain, gait abnormality, and left pelvic mass.	Outside imaging suggested a left ischial tumor.	Synovitis was initially considered; symptoms persisted.
2 November 2025	Admission to the reporting hospital.	Near-normal blood counts and no circulating blasts.	Multidisciplinary evaluation for a primary bone/small round cell tumor.
4 November 2025	Pelvic CT angiography (CTA).	Ill-defined lytic left ischial lesion, approximately 22 mm × 32 mm × 41 mm, with cortical disruption, periosteal reaction, soft-tissue extension, and obturator-region invasion/compression.	Malignant primary bone tumor favored; MRI recommended.
5 November 2025	Pelvic MRI and submission of left ischial tissue biopsy.	Heterogeneous 24 mm × 35 mm × 40 mm lesion with diffusion restriction and enhancement; suspicious right ischial and bilateral iliac lesions. Imaging differential included Ewing/other round cell tumor, metastatic neuroblastoma, Langerhans cell histiocytosis, and osteomyelitis. Biopsy favored a Ewing-like small round cell sarcoma.	Sarcoma-directed diagnostic pathway; molecular testing requested.
12 November 2025	Bone marrow trephine biopsy submitted.	Focal marrow infiltration. Tumor cells: CD61 weak, CD56 strong, WT1/CD43/ERG/CD117/BCL2 positive; lysozyme/MPO/TdT/CD71 negative.	Evidence of systemic involvement and possible megakaryoblastic differentiation.
13 November 2025	Treatment initiated before molecular resolution.	Provisional Ewing-like sarcoma diagnosis.	One cycle of cyclophosphamide, adriavamycin (doxorubicin), and vincristine (CAV) chemotherapy.
During diagnostic work-up	Tissue molecular reclassification.	EWSR1 FISH negative. Broad RNA panel excluded covered Ewing/Ewing-like fusion events and detected CBFA2T3 exon 11::GLIS2 exon 3.	Diagnosis revised to CBFA2T3::GLIS2-positive myeloid sarcoma with focal marrow involvement.
5–14 December 2025	Ten-day myeloid-directed chemotherapy course.	Cytarabine, etoposide, and daunorubicin administered.	Treatment redirected from sarcoma to myeloid disease.
After chemotherapy	Severe marrow suppression.	WBC 0.08 × 10^9^/L, hemoglobin 62 g/L, platelets 23 × 10^9^/L.	Supportive care including meropenem and voriconazole.
After myeloid-directed chemotherapy	Bone marrow reassessment.	Aspirate and trephine biopsy showed no evident tumor involvement; flow cytometry detected no abnormal population.	No detectable marrow involvement was identified using the methods performed; not a formal serial measurable residual disease (MRD) comparison because baseline flow cytometry was unavailable.
After myeloid-directed chemotherapy	Pelvic MRI reassessment.	Marked reduction of the left ischial mass, loss of obvious diffusion restriction, reduced right ischial/bilateral iliac abnormalities, but persistent residual enhancement and bone destruction.	Partial radiologic response.
After transfer to another center	Further individualized treatment.	Available follow-up information indicated targeted therapy including ruxolitinib, revumenib, and lisaftoclax; detailed sequence and doses were unavailable.	Subsequent management outside the reporting institution.
Subsequent follow-up at the receiving center	Maternal haploidentical hematopoietic stem cell transplantation.	Maternal haploidentical hematopoietic stem cell transplantation was performed; the first reported post-transplant molecular reassessment was negative for CBFA2T3::GLIS2.	Early molecular response reported; detailed transplant and long-term follow-up data were unavailable.

### 2.2 Laboratory examinations

The white blood cell count was 9.38 × 10^9^/L, with an absolute neutrophil count of 4.07 × 10^9^/L and an absolute lymphocyte count of 4.54 × 10^9^/L. Hemoglobin was 115 g/L and the platelet count was 193 × 10^9^/L. High-sensitivity C-reactive protein was 20.8 mg/L and procalcitonin was 1.053 ng/mL. No blasts were identified on peripheral blood smear. Lactate dehydrogenase was 208 IU/L and ferritin was 69 ng/mL. The results of microbiologic studies were unremarkable. The absence of cytopenias, leukocytosis, or circulating blasts provided no conventional hematologic entry point and reinforced the initial impression of a localized bone/soft-tissue tumor.

### 2.3 Bone marrow examinations

Bone marrow aspiration showed markedly active hematopoiesis. Granulocytic and erythroid cells comprised 46.5% and 33.5% of nucleated cells, respectively, yielding a myeloid-to-erythroid ratio of 1.39:1. More than 500 megakaryocytes were observed in a 2 cm × 3 cm smear area, and platelets were readily identified. Approximately 2% of nucleated cells were unclassified large cells with round-to-oval, often eccentric nuclei, finely dispersed chromatin, one to three distinct nucleoli, and scant-to-moderate basophilic cytoplasm (**Figure 2A**). The morphology report raised two possibilities: primitive megakaryocytic cells and marrow involvement by an extramedullary tumor. Integrated immunophenotypic, cytogenetic, and fusion-gene assessment was recommended. Because the atypical cells were sparse and not independently lineage-defining, they were interpreted as a diagnostic warning rather than evidence sufficient for AMKL.

**Figure 2:**
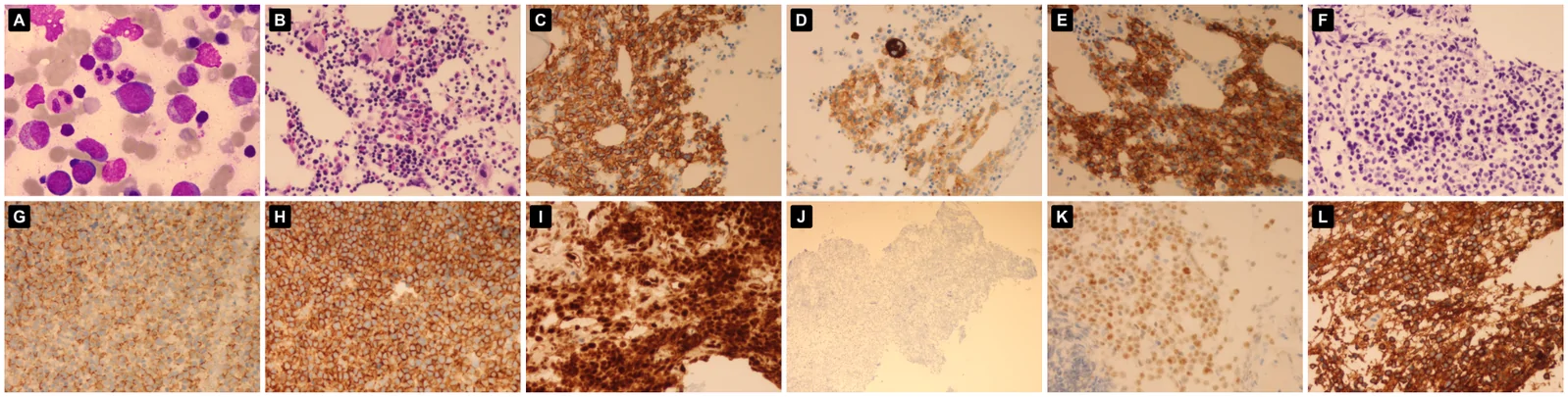
Morphologic and immunohistochemical findings (arranged as six panels per row). **(A)** Bone marrow aspirate showing a large atypical cell with an eccentric round-to-oval nucleus, fine chromatin, a visible nucleolus, and basophilic cytoplasm (Wright–Giemsa stain, original magnification ×1,000). **(B–E)** Bone marrow trephine biopsy showing focal infiltration by atypical tumor cells [**(B)**, hematoxylin and eosin, ×400], with strong membranous CD56 expression [**(C)**, ×400], weak and focal CD61 expression [**(D)**, ×400], and CD117 expression [**(E)**, ×400]. **(F–L)** Left ischial lesion biopsy showing a diffuse proliferation of relatively uniform small round cells [**(F)**, hematoxylin and eosin, ×400]. The tumor cells show membranous CD99 expression [**(G)**, ×400], strong membranous CD56 expression [**(H)**, ×400], nuclear FLI-1 expression [**(I)**, ×400], absence of NKX2.2 expression [**(J)**, ×100], nuclear WT1 expression [**(K)**, ×400], and membranous/cytoplasmic CD43 expression [**(L)**, ×400].

The bone marrow trephine biopsy, submitted on 12 November 2025, showed focal aggregates of small round tumor cells that resembled those in the pelvic lesion (**Figure 2B**). The tumor cells showed weak and focal CD61 and strong CD56 expression and were positive for WT1, CD43, ERG, CD117, and BCL2. Lysozyme, MPO, TdT, and CD71 were negative (**Figures 2C–E**). CD3 and PAX5 labeled only background T and B lymphocytes, respectively. Reticulin and iron stains were negative. Weak CD61 expression alone was insufficient to establish megakaryoblastic lineage; however, the combined pattern of strong CD56, weak CD61, and WT1/CD43/ERG/CD117 expression in a marrow infiltrate morphologically matching the pelvic tumor was discordant with a conventional Ewing sarcoma and raised concern for a CBFA2T3::GLIS2-associated myeloid neoplasm. Bone marrow flow cytometric immunophenotyping was not performed. Accordingly, lineage assessment in the marrow was based on aspirate morphology and trephine-biopsy immunohistochemistry rather than a multiparameter RAM-profile evaluation.

### 2.4 Histopathological and immunohistochemical examinations

The left ischial lesion biopsy was submitted on 5 November 2025. Histological examination showed diffuse bone infiltration by relatively uniform small round cells with clear cytoplasm and finely textured chromatin (**Figure 2F**). The initial interpretation was an undifferentiated small round cell sarcoma with Ewing-like features, driven by the destructive pelvic bone lesion, monomorphic round cell morphology, and tumor-cell expression of CD99 and FLI-1. Tumor cells were also positive for CD56, CD43, CD117, ERG, BCL2, INI1, BRG1, and WT1, with scattered vimentin expression. They were negative for ETV4, NKX2.2, pancytokeratin, chromogranin A, epithelial membrane antigen, SATB2, calretinin, synaptophysin, TLE1, CD45, desmin, BCOR, MyoD1, myogenin, S100, PHOX2B, MPO, pan-TRK, CD19, TdT, and CD20. Rare cells expressed CD34, and the hotspot Ki-67 proliferation index was approximately 30% (**Figures 2G–L**). CD99 and FLI-1 are not lineage-specific, whereas the absence of NKX2.2 and a broad negative panel argued against classic Ewing sarcoma, rhabdomyosarcoma, neuroblastoma, lymphoblastic neoplasia, and several other pediatric small round cell tumors. Conversely, strong CD56 together with CD43, CD117, WT1, and ERG expression provided a myeloid-associated signal that required correlation with the marrow findings and fusion testing. Two external pathology consultations also prioritized a Ewing-like small round cell sarcoma and recommended molecular testing.

### 2.5 Molecular examinations

All molecular analyses were performed on pelvic lesion tissue; pretreatment marrow was not tested molecularly. EWSR1 break-apart FISH showed fused signals in 91% of nuclei and split signals in 9%. Because the split-signal proportion was below the laboratory cutoff of 15%, the result was interpreted as negative (**Figure 1A**). This negative result did not by itself exclude rare FUS::ETS-positive Ewing sarcoma or other genetically defined round cell sarcomas. Therefore, broad tissue RNA fusion sequencing was performed and included targets relevant to Ewing sarcoma and Ewing-like entities. No covered sarcoma-defining fusion was detected. Instead, the assay identified an in-frame CBFA2T3::GLIS2 fusion joining CBFA2T3 exon 11 to GLIS2 exon 3 (**Figure 1B**). When integrated with the focal marrow infiltrate and its strong CD56, weak CD61, and WT1/CD43/ERG/CD117 profile, this result provided the decisive basis for revising the diagnosis from a Ewing-like sarcoma to a CBFA2T3::GLIS2-positive myeloid neoplasm.

### 2.6 Diagnosis and differential diagnosis

The integrated diagnosis was CBFA2T3::GLIS2-positive myeloid sarcoma with focal bone marrow involvement and supportive evidence of megakaryoblastic differentiation, within the biological spectrum of CBFA2T3::GLIS2-positive AMKL. The diagnostic revision was not based on the fusion result in isolation. The marrow aspirate first identified a small population of unexplained atypical cells; a trephine biopsy then established focal systemic involvement by a tumor morphologically concordant with the pelvic lesion. Immunohistochemistry demonstrated strong CD56, weak CD61, and WT1/CD43/ERG/CD117 expression; and broad RNA testing excluded a covered Ewing/Ewing-like driver while detecting CBFA2T3::GLIS2. This formulation also reflects the limits of the available evidence: the marrow aspirate contained only approximately 2% atypical cells, marrow involvement was focal, CD61 expression was weak, diagnostic marrow flow cytometry was not performed, and the fusion was demonstrated in tissue rather than independently in marrow. Collectively, however, the concordant tissue, marrow, immunophenotypic, and molecular findings supported a myeloid neoplasm with megakaryoblastic differentiation (5–13).

The principal differential diagnoses and the evidence used to distinguish them from a CBFA2T3::GLIS2-positive myeloid neoplasm are summarized in **Table 2**.

**Table 2:** Differential diagnosis of the Ewing-like pelvic lesion.

Entity	Overlapping features	Evidence resolving the diagnosis
Classic Ewing sarcoma (14–17)	Pelvic bone lesion, lytic destruction, soft-tissue mass, restricted diffusion, uniform small round cells, and CD99/FLI-1 expression.	NKX2.2 negative; EWSR1 break-apart FISH negative; broad RNA assay did not detect an EWSR1::ETS or FUS::ETS fusion; focal marrow infiltration with strong CD56 and weak CD61; tissue CBFA2T3::GLIS2 fusion.
CIC-, BCOR-, or other Ewing-like round cell sarcoma (16, 17)	Undifferentiated small round cell morphology and variable CD99 expression.	BCOR and ETV4 negative; broad RNA assay did not detect a sarcoma-defining fusion covered by the panel and instead identified CBFA2T3::GLIS2.
Rhabdomyosarcoma	Infantile soft-tissue mass with small round cell morphology.	Desmin, MyoD1, and myogenin negative.
Lymphoblastic lymphoma/leukemia	Small round cell tumor with marrow involvement.	TdT, CD19, and CD20 negative; PAX5 and CD3 labeled only background lymphocytes.
Neuroblastoma/other neuroendocrine tumor	Infant age, bone lesion, possible multifocal marrow/osseous disease, and CD56 expression.	PHOX2B, synaptophysin, and chromogranin A negative; marrow CD61/WT1/CD43/ERG/CD117 profile and tissue CBFA2T3::GLIS2 support myeloid disease.
Langerhans cell histiocytosis	Infant age, lytic bone lesion, soft-tissue extension, and possible multifocal osseous abnormalities.	Monomorphic blast-like morphology without typical grooved histiocytes or an eosinophil-rich background; S100 negative; marrow CD61/WT1/CD43/ERG/CD117 profile and tissue CBFA2T3::GLIS2 support myeloid disease.
Osteomyelitis/bone abscess	Pain, elevated inflammatory markers, osteolysis, periosteal reaction, surrounding edema, and heterogeneous enhancement.	Microbiologic studies were unremarkable; biopsy demonstrated a clonal small round cell neoplasm; marrow infiltration and CBFA2T3::GLIS2 fusion established malignant myeloid disease.
CBFA2T3::GLIS2-positive myeloid sarcoma with focal marrow involvement (12, 13)	Can form destructive bone/soft-tissue masses with small round cell morphology, CD45/MPO negativity, strong CD56, and variable megakaryocytic-marker expression.	Focal marrow infiltration; CD61 weak; CD56 strong; WT1/CD43/ERG/CD117/BCL2 positive; EWSR1-negative tissue; broad RNA assay excluded a covered Ewing/Ewing-like driver and detected CBFA2T3::GLIS2; most consistent.

### 2.7 Treatment and follow-up

Because the clinical, radiologic, and biopsy findings initially favored a primary small round cell sarcoma, the patient received one cycle of cyclophosphamide, adriavamycin (doxorubicin), and vincristine (CAV) chemotherapy on 13 November 2025 before molecular resolution. Once EWSR1 FISH was negative and broad RNA testing identified CBFA2T3::GLIS2 in the setting of concordant focal marrow involvement, the disease was reclassified as a systemic myeloid neoplasm rather than a localized Ewing-family sarcoma. Sarcoma-directed therapy was therefore discontinued, and treatment was redirected to AML-type induction intended to address both marrow and extramedullary disease. Beginning on 5 December 2025, the patient received a 10-day course comprising cytarabine, etoposide, and daunorubicin. The course was followed by severe myelosuppression, with a white blood cell count of 0.08 × 10^9^/L, hemoglobin of 62 g/L, and a platelet count of 23 × 10^9^/L. Supportive and anti-infective management included meropenem and voriconazole.

On post-treatment reassessment, bone marrow aspirate morphology and trephine biopsy showed no evident tumor-cell involvement. Post-treatment multiparameter flow cytometry did not identify a phenotypically abnormal cell population. Because no flow cytometric immunophenotyping had been performed at diagnosis, this negative post-treatment result could not be interpreted as conventional longitudinal measurable residual disease assessment against a defined baseline leukemia-associated immunophenotype.

Follow-up pelvic MRI, compared with the examination of 5 November 2025, showed marked shrinkage of the previously large left ischial soft-tissue mass (**Figure 1D**). The residual lesion remained heterogeneous, predominantly low signal on T1-weighted imaging and high signal on T2-Dixon water images, but no longer showed obvious diffusion restriction. Heterogeneous enhancement persisted with small non-enhancing areas. Residual abnormalities still involved the ischiopubic junction and superior pubic ramus with persistent bone destruction, although the extent and signal abnormality were reduced. Compression of the adjacent bladder and obturator internus was no longer evident, and the previously enlarged lymph node between the lesion and the left bladder wall was no longer visualized. Suspicious abnormalities in the right ischium and bilateral iliac bones also decreased in extent and signal intensity. These findings were consistent with a partial radiologic response rather than complete resolution.

The patient was subsequently transferred to another center for continued treatment. Available follow-up documentation indicated that management included individualized targeted agents—ruxolitinib, revumenib, and lisaftoclax—followed by maternal haploidentical hematopoietic stem cell transplantation. The receiving center’s formal decision record was unavailable; nevertheless, proceeding to allogeneic transplantation was clinically consistent with the adverse-risk biology and chemoresistance associated with CBFA2T3::GLIS2, the need to consolidate systemic disease after response, and contemporary practice for high-risk pediatric AML (19). Recent experimental work has identified JAK2 dependence and ruxolitinib sensitivity in CBFA2T3::GLIS2-positive models, but this strategy remains investigational (20). The rationale and contribution of revumenib and lisaftoclax could not be reconstructed from the available records, and no efficacy is attributed to any individual agent. The first reported molecular reassessment after transplantation showed no detectable CBFA2T3::GLIS2 fusion transcript. Because treatment and follow-up were performed outside the reporting institution, the exact sequencing and dosing of these agents, transplant conditioning and graft-versus-host disease prophylaxis, molecular assay parameters, and longer-term outcomes were not available. These post-transfer data are therefore presented as a high-level clinical summary.

## 3 Discussion

This case illustrates a stepwise diagnostic revision rather than a diagnosis established by any single test. First, the infant’s destructive ischial lesion, substantial soft-tissue component, restricted diffusion, uniform small round cell histology, and CD99/FLI-1 expression strongly favored Ewing sarcoma and created a risk of diagnostic anchoring. Second, approximately 2% unexplained large cells in an otherwise maturing marrow prompted trephine biopsy, which confirmed focal infiltration by a tumor morphologically concordant with the pelvic lesion. Third, strong CD56, weak CD61, and WT1/CD43/ERG/CD117 expression introduced a myeloid/megakaryoblastic signal that was not explained by Ewing sarcoma. Fourth, EWSR1 break-apart FISH was negative. Finally, broad RNA fusion testing excluded a covered Ewing/Ewing-like driver and identified CBFA2T3::GLIS2. The critical pitfall was over-weighting anatomic site, round cell morphology, CD99, and FLI-1 while under-weighting the patient’s age, marrow findings, and the non-lineage-specific nature of these markers (12–17).

The immunophenotypic differential diagnosis deserves particular emphasis. CD99 and FLI-1 are sensitive but not specific for Ewing sarcoma, and NKX2.2 was negative in this case (14, 15). The absence of desmin, MyoD1, and myogenin argued against rhabdomyosarcoma; PHOX2B, synaptophysin, and chromogranin A negativity argued against neuroblastoma; TdT and B-lineage marker negativity argued against lymphoblastic neoplasia; and the morphology and S100 negativity did not support Langerhans cell histiocytosis. Conversely, CBFA2T3::GLIS2-positive AMKL frequently shows a RAM or RAM-like phenotype characterized by bright CD56, diminished CD45 and CD38, absent HLA-DR, and variable CD34, CD33, CD117, CD41a, CD61, and CD42b (8–11). Diagnostic marrow flow cytometry was not performed, so a formal RAM phenotype cannot be assigned. Weak CD61 alone was also not lineage-defining. Its significance emerged only when combined with strong CD56, WT1/CD43/ERG/CD117 expression, focal marrow infiltration, and the defining fusion (5–13).

The low-level marrow abnormality warranted attention without overinterpretation. Approximately 2% atypical cells could not establish AMKL, but their morphology was discordant with the otherwise maturing marrow and generated a testable hypothesis of primitive megakaryocytic cells or marrow involvement by an extramedullary tumor. Trephine biopsy then confirmed focal infiltration. Thus, morphology provided the trigger, immunohistochemistry established concordant marrow disease, and molecular testing resolved lineage and entity. This sequence demonstrates the added value of parallel tissue and marrow evaluation in an infantile small round cell tumor, even when peripheral blood counts are near normal and circulating blasts are absent.

The absence of pretreatment marrow flow cytometry and paired marrow molecular testing remains a material limitation. A RAM-oriented flow panel would have assessed CD56, CD45, CD38, HLA-DR, CD33, CD117, CD34, CD36, and platelet glycoproteins CD41a, CD61, and CD42b (7–13). Paired detection of CBFA2T3::GLIS2 in marrow and tissue would also have provided direct molecular proof of clonal identity. Post-treatment flow cytometry identified no abnormal population, but the lack of a diagnostic leukemia-associated immunophenotype prevented conventional longitudinal measurable residual disease assessment. The diagnosis therefore rests on convergence across four domains—morphology, tissue/marrow immunophenotype, marrow distribution, and broad RNA fusion testing—rather than on any isolated marker.

Molecular testing was decisive and also placed the case in a recognized biological spectrum. CBFA2T3::GLIS2 results from a cryptic chromosome 16 inversion, dysregulates transcriptional programs linked to self-renewal and megakaryoblastic differentiation, and is associated with induction resistance, relapse, and poor survival (5–7). Malik et al. described four CBFA2T3::GLIS2-positive myeloid sarcomas that mimicked pediatric small round cell tumors, frequently showing strong CD56, absent or weak conventional myeloid markers, and variable megakaryocytic-marker expression (12). Lebedeva et al. reported an extramedullary AMKL relapse misclassified as secondary Ewing sarcoma until integrated flow and molecular analysis established the correct diagnosis (13). The present case extends this diagnostic spectrum by documenting a *de novo* destructive bone presentation in an infant with only focal marrow involvement. Negative EWSR1 FISH alone was insufficient because rare Ewing sarcomas harbor FUS::ETS fusions, and other genetically defined round cell sarcomas remain possible (16, 17). Broad RNA fusion testing was therefore more informative than serial single-gene assays: it simultaneously failed to identify a covered sarcoma driver and identified the defining myeloid fusion (18).

Diagnostic revision had immediate therapeutic consequences. CAV was initiated under the provisional sarcoma diagnosis, but a CBFA2T3::GLIS2-positive systemic myeloid neoplasm required AML-directed therapy capable of treating both marrow and extramedullary disease. The subsequent cytarabine-, etoposide-, and daunorubicin-based course was followed by clearance of evident marrow disease and a marked, although incomplete, radiologic response. Because CBFA2T3::GLIS2 defines an adverse-risk pediatric AML subgroup, allogeneic hematopoietic stem cell transplantation (HSCT) may be considered as consolidation when remission is achieved. Contemporary Children’s Oncology Group data support HSCT in first remission for high-risk pediatric AML overall, although the magnitude of benefit specifically for CBFA2T3::GLIS2 remains uncertain (19). Targeted therapy for this fusion is not established. JAK2 inhibition has a biological and preclinical rationale, including selective sensitivity to ruxolitinib in experimental models, but remains investigational (20). The externally administered drug sequence and transplant details were incomplete, so this case cannot establish efficacy for ruxolitinib, revumenib, lisaftoclax, haploidentical transplantation, or any specific sequence. Its principal contribution is diagnostic and therapeutic triage: correct molecular classification redirected management from a sarcoma pathway to high-risk myeloid therapy.

In conclusion, CBFA2T3::GLIS2-positive myeloid neoplasia should be considered in infantile Ewing-like bone and soft-tissue tumors, particularly when CD56 is strong, myeloid-associated markers are present, marrow contains low-level atypical cells, or EWSR1 testing is negative (12, 13). The most defensible diagnostic pathway is parallel rather than sequential: review tissue morphology and immunophenotype, examine marrow even when blood counts are near normal, avoid treating CD99 or FLI-1 as lineage-specific, and use broad RNA fusion testing when targeted sarcoma assays are negative or discordant. In this patient, the combination of focal marrow infiltration, weak CD61 expression, a myeloid-associated immunophenotypic pattern, exclusion of a coherent Ewing/Ewing-like fusion, and detection of CBFA2T3::GLIS2 supported diagnostic revision and timely myeloid-directed management. Baseline flow cytometry, paired marrow molecular testing, and complete external treatment records would have strengthened lineage, clonal, residual-disease, and therapeutic interpretation.

## Data Availability

The original contributions presented in the study are included in the article. Further inquiries can be directed to the corresponding author.
